# Pathophysiology of skeletal muscle disturbances in Myalgic Encephalomyelitis/Chronic Fatigue Syndrome (ME/CFS)

**DOI:** 10.1186/s12967-021-02833-2

**Published:** 2021-04-21

**Authors:** Klaus J. Wirth, Carmen Scheibenbogen

**Affiliations:** 1 KOSA Pharma GmbH, Frankfurt am Main, Germany; 2 Institute of Medical Immunology, Charité - Universitätsmedizin Berlin, Corporate Member of Freie Universität Berlin, Humboldt-Universität zu Berlin, and Berlin Institute of Health, Berlin, Germany

**Keywords:** Chronic Fatigue Syndrome, Myalgic Encephaloymelitis, ß2-adrenergic receptor, Mitochondrial dysfunction, Na^+^/K^+^-ATPase, Sodium-proton-exchanger, Sodium-calcium-exchanger, Post-acute COVID-19 syndrome

## Abstract

Chronic Fatigue Syndrome or Myalgic Encephaloymelitis (ME/CFS) is a frequent debilitating disease with an enigmatic etiology. The finding of autoantibodies against ß2-adrenergic receptors (ß2AdR) prompted us to hypothesize that ß2AdR dysfunction is of critical importance in the pathophysiology of ME/CFS. Our hypothesis published previously considers ME/CFS as a disease caused by a dysfunctional autonomic nervous system (ANS) system: sympathetic overactivity in the presence of vascular dysregulation by ß2AdR dysfunction causes predominance of vasoconstrictor influences in brain and skeletal muscles, which in the latter is opposed by the metabolically stimulated release of endogenous vasodilators (functional sympatholysis). An enigmatic bioenergetic disturbance in skeletal muscle strongly contributes to this release. Excessive generation of these vasodilators with algesic properties and spillover into the systemic circulation could explain hypovolemia, suppression of renin (paradoxon) and the enigmatic symptoms. In this hypothesis paper the mechanisms underlying the energetic disturbance in muscles will be explained and merged with the first hypothesis. The key information is that ß2AdR also stimulates the Na^+^/K^+^-ATPase in skeletal muscles. Appropriate muscular perfusion as well as function of the Na^+^/K^+^-ATPase determine muscle fatigability. We presume that dysfunction of the ß2AdR also leads to an insufficient stimulation of the Na^+^/K^+^-ATPase causing sodium overload which reverses the transport direction of the sodium-calcium exchanger (NCX) to import calcium instead of exporting it as is also known from the ischemia–reperfusion paradigm. The ensuing calcium overload affects the mitochondria, cytoplasmatic metabolism and the endothelium which further worsens the energetic situation (vicious circle) to explain postexertional malaise, exercise intolerance and chronification. Reduced Na^+^/K^+^-ATPase activity is not the only cause for cellular sodium loading. In poor energetic situations increased proton production raises intracellular sodium via sodium-proton-exchanger subtype-1 (NHE1), the most important proton-extruder in skeletal muscle. Finally, sodium overload is due to diminished sodium outward transport and enhanced cellular sodium loading. As soon as this disturbance would have occurred in a severe manner the threshold for re-induction would be strongly lowered, mainly due to an upregulated NHE1, so that it could repeat at low levels of exercise, even by activities of everyday life, re-inducing mitochondrial, metabolic and vascular dysfunction to perpetuate the disease.

## Background

Chronic Fatigue Syndrome or Myalgic Encephaloymelitis (ME/CFS) is a debilitating disease with a world-wide prevalence of 0.2–0.5% and is often triggered by various viral infections among them EBV, enteroviruses, influenza, dengue fever and others [1]. ME/CFS is characterized by profound fatigue, exertional intolerance, sleep disturbance, muscle pain, orthostatic intolerance and various other symptoms. The cardinal symptom is exertional intolerance with post-exertional malaise (PEM), a disproportionate aggravation of symptoms after physical or mental effort [2]. Chronic muscle pain and weakness is a typical symptom of ME/CFS which can increase for days after minor exertion. Assessment of hand grip strength shows both impaired strength and enhanced fatiguability in ME/CFS and correlates well with disease severity, muscle pain and PEM [3]. Diagnosing of ME/CFS is challenging for patients and physicians due to many unspecific symptoms, the broad differential diagnosis of chronic fatigue and the lack of an established biomarker. So far, ME/CFS is a clinical diagnosis classified as neurological disease by the WHO with the international Canadian Consensus Criteria (CCC) as the most accepted [1].

**Fig. 1:**
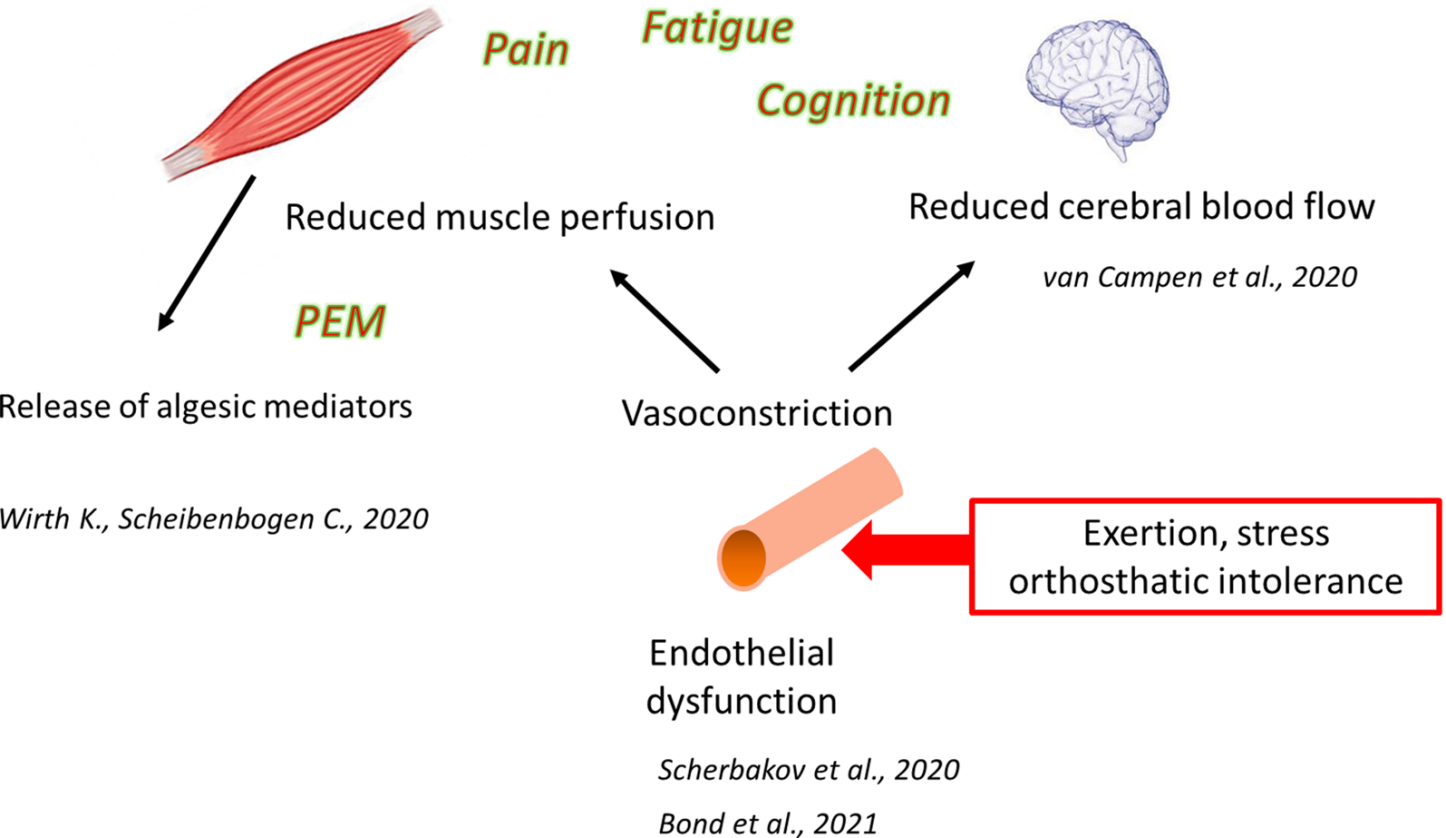
Key symptoms of ME/CFS related to hypoperfusion

To date, the etiology and pathophysiology of ME/CFS is still unresolved, but there is ample evidence for autonomic and vascular dysregulation [4–6]. Hypoperfusion of muscles and impaired cerebral blood flow upon exertion are considered as key mechanisms for fatigue muscle pain, PEM and impaired cognition [6–10] (Fig. 1). Further, there is evidence for autoimmunity and natural autoantibodies against ß2-adrenergic receptors (ß2AdR) and M3-muscarinergic receptors were found upregulated in about one third of the patients and the function of ß2AdR antibodies was found to be attenuated in a subsequent study [11–13]. A recent study showed a correlation between levels of ß2AdR antibodies and brain network alterations associated with pain [14]. Immuno adsorption removing IgG resulted in improvement of the disease [15]. These antibodies belong to a of natural antibodies against adrenergic, cholinergic and other GPCR receptors which is dysregulated in various autoimmune diseases [16]. These findings prompted us to deeper consider the possibility that dysfunction of ß2AdR could play a major role in the pathophysiology of ME/CFS [6]. To strengthen the argument of ß2AdR dysfunction, adolescent ME/CFS was found associated with polymorphisms of the ß2AdR gene [17]. Mutations of this receptor show a cardiovascular profile in healthy adults which can be considered an asymptomatic form of the cardiovascular changes found in ME/CFS [18, 19]. ß2AdR are particularly sensitive to desensitization by chronic sympathetic stimulation evident in ME/CFS [20, 21]. The propensity for agonist induced desensitization is even enhanced in subjects with mutant ß2AdR [18]. It was reported that ME/CFS patients had lower expression of ß2AdR [22]. Altogether, autoantibodies, mutations and desensitization may cause dysfunction of the ß2AdR and act synergistically with other causes of endothelial dysfunction (ED) induced by the viral infection.

In a previous paper entitled “A Unifying Hypothesis of the Pathophysiology of Myalgic Encephalomyelitis/Chronic Fatigue Syndrome (ME/CFS): Recognitions from the finding of autoantibodies against ß2-adrenergic receptors “we discussed how ß2AdR dysfunction could be causally involved in the pathophysiology of ME/CFS [6]. To briefly summarize this hypothesis: ß2AdR are important vasodilator receptors in the heart, brain and skeletal muscles, and are expressed on endothelial cells to release nitric oxide and on vascular smooth muscles cells to cause direct vasodilation. M3-muscarinergic receptors release nitric oxide from endothelial cells. The dysfunction of these receptors may result in ED impairing vasodilator influences. Mental and skeletal muscle fatigue that are hallmarks of ME/CFS may be caused by ED resulting in misery perfusion. Along with this vascular disturbance a unique cardiovascular situation is found in ME/CFS that is characterized by hypovolemia, preload failure, small hearts, orthostatic intolerance, postural tachycardia (POTS), chronotropic incompetence and most surprisingly a paradoxically low activity of the renin–angiotensin–aldosterone system. Numerous heart rate variability studies show a high sympathetic (vasoconstrictor) tone and a low vagal tone. Altogether, vasoconstrictor influences seem to be increased while vasodilator effects are obviously impaired by ß2AdR dysfunction and/or ED of other causes mentioned above.

Our hypothesis considers ME/CFS as a disease of a dysfunctional autonomic nervous system (ANS) resulting from sympathetic overactivity in the presence of dysfunctional ß2AdR and ED. The combined disturbance leads to a disruption of the physiological interaction between skeletal muscles and the cardiovascular system. High sympathetic tone in the presence of vascular dysfunction may lead to an excessive vasoconstrictor stimulus in the brain and skeletal muscles, which in the latter is counter-regulated by the metabolically stimulated generation of endogenous vasodilators in the physiological process of functional sympatholysis. A still enigmatic bioenergetic disturbance in skeletal muscle [23] strongly enhances the metabolically stimulated compensatory release of endogenous vasodilators in the muscle. Some of the released vasodilators like bradykinin and prostaglandins have algesic, hyperalgesic and spasmogenic actions and cause microvascular leakage and even open the blood brain barrier. Excessive generation of algesic vasodilators and their spillover into the systemic circulation could explain the unfavorable CV-situation causing hypovolemia and preload failure by microvascular leakage, renal hyperexcretion and by inhibition of renin production as consequence of renal hyperemia and tubular effects induced by those vasodilators. The latter annihilate the physiological signals for renin generation so that renin remains low and re-filling of the vasculature does not take place to perpetuate the state of hypovolemia. This in turn further raises sympathetic tone to even cause more vasoconstriction leading to a vicious circle. The appearance of hyperalgesic and spasmogenic vasodilators in the systemic circulation may explain many of the enigmatic symptoms of ME/CFS including pain, hyperalgesia, intestinal complaints, flu-like symptoms, sore lymph nodes and others.

In this second hypothesis paper the presumed mechanisms underlying the energetic disturbance in skeletal muscles in ME/CFS will be discussed. Various studies provide evidence of impaired muscle function and metabolism in ME/CFS. Patients with ME/CFS perform worse than controls in a controlled repeated exercise study [24]. Cultured human skeletal muscle cells from patients with ME/CFS show impaired glucose uptake and ATP levels [25]. Skeletal muscle acidosis or dysregulation of protons is found in ME/CFS patients during or after exercise [23, 26–29]. A diminished oxygen supply of muscles upon exercise is shown in several studies in ME/CFS patients [24], 30. In line with this, metabolic changes in ME/CFS indicate hypoxia and ischemia [31].

While in the previous hypothesis paper we had focused on the role of ß2AdR as important vasodilators in the brain and skeletal muscle and on their role in the heart (positive chronotropic and inotropic effects) in this paper we focus on the potential pathomechanistic role of the ß2AdR as an important stimulator of the Na^+^/K^+^-ATPase in skeletal muscles [32, 33]. Appropriate muscular perfusion as well as function of the Na^+^/K^+^-ATPase activity determine muscle fatigability. Experimentally, the function of Na^+^/K^+^-ATPase is strongly related to recovery of muscular force [32]. We presume that dysfunction of the ß2AdR in the presence of a high sympathetic tone not only leads to impaired muscular perfusion but also to an insufficient stimulation of the Na^+^-K^+^-ATPase and that this entails complex ionic disturbances in the muscles, which particularly affect the mitochondria, to further worsen the energetic situation and cause exercise intolerance and postexertional malaise (PEM).

## Disturbances of ions in skeletal muscles in ME/CFS as the main mechanism of PEM and exercise intolerance

### Physiological regulation of intracellular ion homeostasis in muscle cells

Sodium enters the myocytes in the process of excitation contraction coupling via sodium channels and via activity of the sodium-proton exchanger subtype1 (NHE1). The latter exports one proton for which the driving force is the import of one sodium ion tending to load the cell with sodium. The NHE1 as the most important proton transporter [34–36] is dependent on the sodium gradient as the driving force for proton extrusion (and not directly on ATP). The physiological sodium gradient that drives the NHE1is maintained by the Na^+^/K^+^-ATPase. Sodium is removed from the cell by the Na^+^/K^+^-ATPase at the expense of ATP consumption to maintain excitability and the sodium gradient as a driving force for transport processes. Na^+^/K^+^-ATPase is electrogenic exporting three sodium ions in exchange for the import of two potassium ions.

### Disturbances of intracellular ions in ME/CFS

**Fig. 2:**
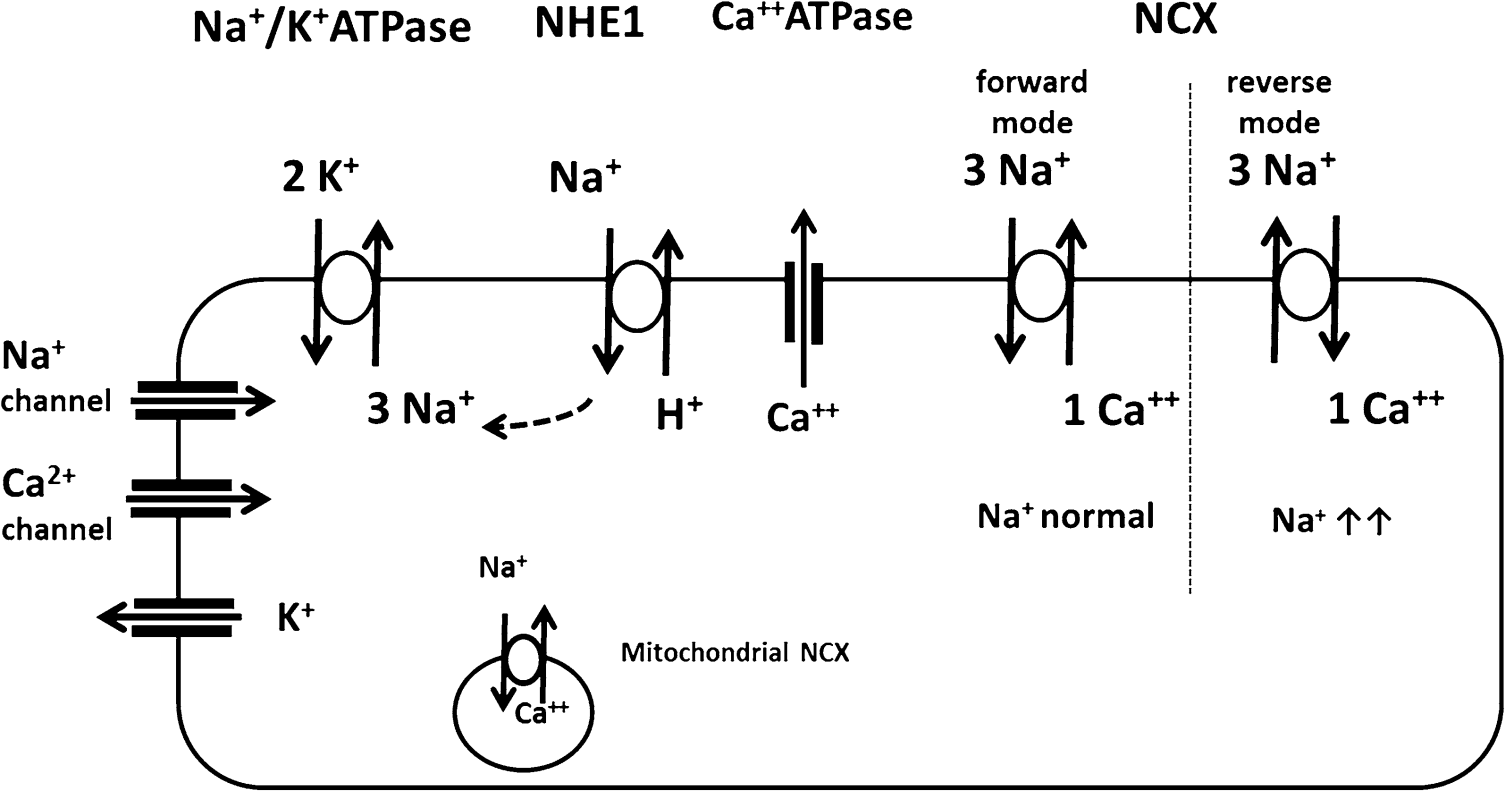
Transporters and channels involved in the handling of protons, sodium, calcium and potassium in myocytes. At normal sodium concentrations the NCX of the sarcolemma works in the forward mode to export calcium. At high sodium concentrations caused by high NHE1- and low Na^+^/K^+^-ATPase activity the NCX reverses its transport direction to import calcium leading to calcium overload. Since intracellular sodium is the driving force for the mitochondrial NCX, elevated intracellular sodium (even below the threshold for reverse mode of the sarcolemmal NCX) activates the mitochondrial NCX to diminish mitochondrial calcium while elevated cytoplasmatic calcium has the opposing effect and tends to increase it. KATP is closed at normal but activated at low ATP levels. Opening will cause hyperpolarization, a protective mechanism against calcium overload. But if open for a longer time it can cause potassium loss together with the weak Na^+^/K^+^-ATPase activity, for which the causes are listed in Table 1. Intracellular hypokalemia reduces the driving force of the protective KATP opening

Disturbances of ionic transport are well known from experimental ischemia–reperfusion injury situations in the heart and in skeletal muscle. Harmful ionic disturbances appear abruptly within a minute of reperfusion after a period of total ischemia whether it is the heart or skeletal muscle. Although ME/CFS is not an ischemia–reperfusion situation we provide arguments that similar ionic disturbances (the ischemia–reperfusion injury paradigm) could occur in skeletal muscles of ME/CFS patients but would appear more slowly and be less severe. These ionic changes in skeletal muscles finally result in calcium overload to cause mitochondrial dysfunction, muscular insulin resistance and vascular (endothelial) dysfunction in muscles with repeated exercise. Major players involved are the sodium–potassium ATPase (Na^+^/K^+^-ATPase), the sodium-proton-exchanger (NHE1; SLC9A1) and the sodium-calcium-exchanger (NCX) (Fig. 2). The isoform in skeletal muscle is NCX_3_ (SLC8A3) [37]. The NCX can reverse its transport direction to import calcium instead of exporting it under conditions of a strong rise in intracellular sodium. In the following we try to work out how sodium overload could occur in ME/CFS which would then entail calcium overload as the final damaging mechanism.

### What are the mechanisms that could cause sodium loading in ME/CFS?

In the working muscles, particularly in hypoxia, the increase in anaerobic metabolism should raise proton generation and therefore also cellular sodium load via the **NHE1**. Previous exercise deteriorated physical performance and increased arterial lactate during exercise in patients with ME/CFS while it lowered lactate in healthy subjects [38]. Under conditions of insufficient ATP production **Na**^**+**^**/K**^**+**^**-ATPase** may not be able to remove sodium in sufficient quantities, and intracellular sodium may therefore accumulate. In experimental situations ß2AdR activation stimulates the Na^+^/K^+^-ATPase activity [32] and improves muscle force recovery and so do insulin and calcitonin-gene related peptide (cGRP). Dysfunctional ß2AdR may be a major cause in the pathophysiology of muscular fatigue affecting not only perfusion and the energetic situation but also impairing the stimulation of the Na^+^/K^+^-ATPase. If insulin resistance occurred as a consequence of the metabolic changes of skeletal muscle in ME/CFS, as will be outlined below, the Na^+^/K^+^-ATPase activity would be further impaired.

In skeletal muscle Na^+^/K^+^-ATPase is stimulated by ß2AdR and cGRP, probably during exercise, and by insulin and acetylcholine at rest [32, 33, 39]. We strongly assume that the function of the Na^+^/K^+^-ATPase is severely disturbed in ME/CFS, not only by dysfunctional ß2AdR but also by low ATP production, insulin resistance (explained below) and lowered cGRP production. cGRP-release could be lower for two reasons: (1) lowered expression of the cation channel TRPM3, which could be a predisposing factor. (2) small fiber neuropathy which could develop as a consequence of ME/CFS or be present before as a risk factor [40]. Expression of the cation channel TRPM3 has been found reduced in leukocytes of patients with ME/CFS [41]. It is unknown and remains to be explored whether reduced expression of TRPM3 is also present in small fibres in skeletal muscles. Activation of TRPM3 induces vasodilation of resistance arteries by releasing cGRP und Substance P from perivascular nerves [42] so that this mechanism of vasodilation may be impaired enhancing the need for the metabolically stimulated release of hyperalgesic vasodilators as compensation for this deficit. Impaired cGRP release would also reduce the stimulation of the Na^+^/K^+^-ATPase during exercise increasing the risk of sodium overload as mentioned above. It remains to be investigated whether there is an interaction between ß2AdR dysfunction and possible TRPM3 deficiency, i.e. whether both disturbances are required for ME/CFS to develop or whether a dual disturbance is related to disease severity. The potential disturbances of the Na^+^/K^+^-ATPase in skeletal muscles in ME/CFS are shown in Table 1.

**Table 1:** Activation of Na^+^/K^+^-ATPase by physiological transmitters and ATP in skeletal muscle: possible dysfunction and modulation in ME/CFS

Mechanism	Importance	Na^+^/K^+−^ATPase function in ME/CFS
Acetylcholine	At rest	Enhanced by physostigmine (Mestinon™)
Insulin	At rest	Decreased by potential insulin resistance
ß2AdR-agonist	Exercise	Decreased by ß2AdR-dysfunction/downregulation
ccGRP	Exercise	Decreased by small fiber degeneration Diminished due to low expression of TRPM3?
ATP	Exercise mainly	Decreased due to ATP depletion
ROS	Exercise mainly	Inhibition

**Fig. 3:**
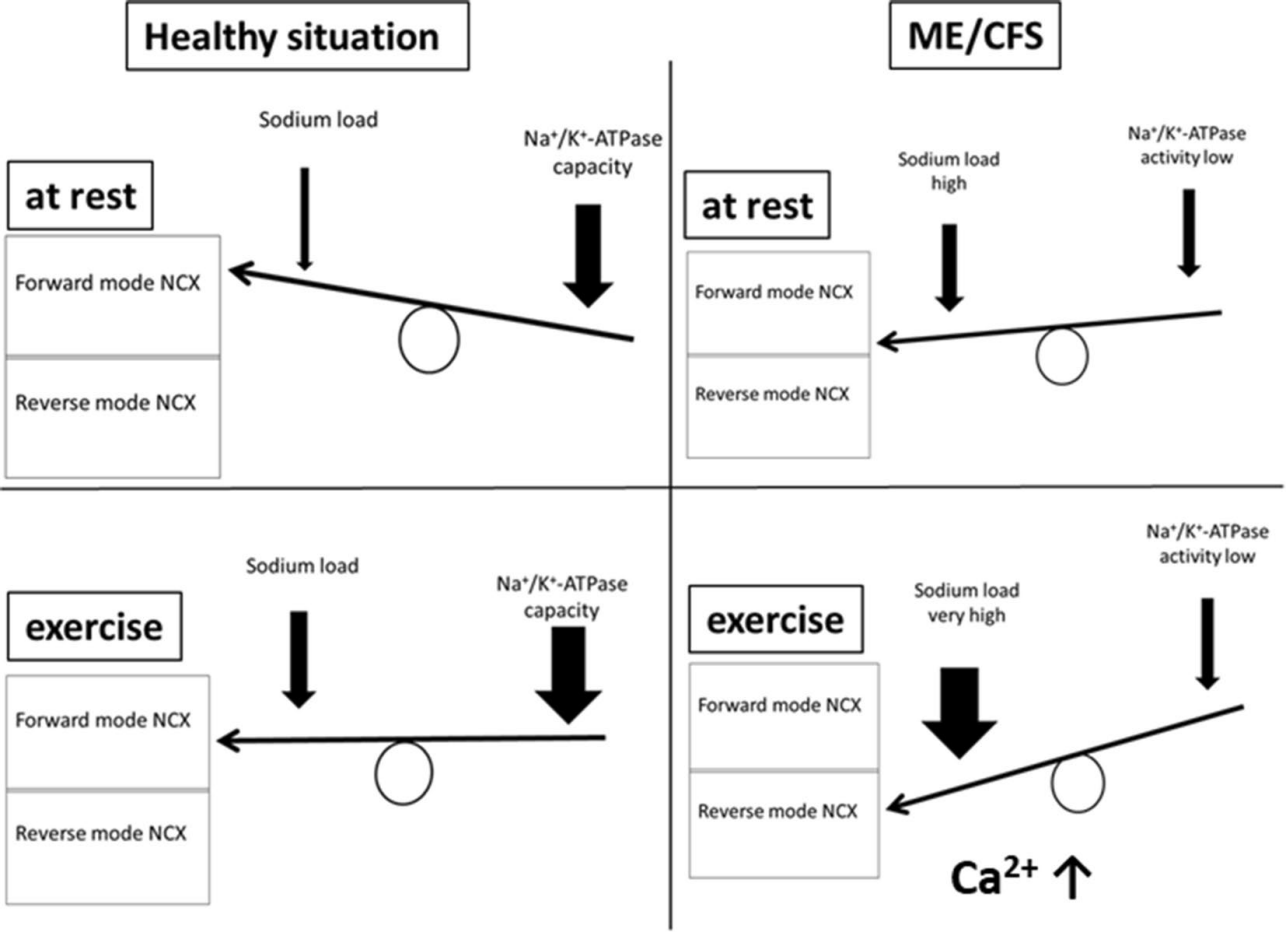
Presumed sodium load in ME/CFS at rest and during exercise in skeletal muscles. Intracellular sodium concentration in striated muscle in ME/CFS is presumably close to the threshold for reverse mode activation of the NCX or at least shows a very rapid rise at even low levels of exercise. This treshold is then rapidly crossed during exercise causing calcium overload which leads to mitochondrial and vascular dysfunction and muscular insulin resistance. The proximity of intracellular sodium concentration to this threshold is the presumed main cause for exercise intolerance in ME/CFS and the potential explanation for the characteristics of a threshold of effort for the induction of PEM

Due to these stimulatory deficits Na^+^/K^+^-ATPase cannot sufficiently clear increased intracellular sodium as we will outline below (imbalance between high sodium influx by NHE1 and low activity of the Na^+^/K^+^-ATPase) leading to sodium overload (Fig. 3). There is also evidence for an inhibition of the Na^+^/K^+^-ATPase by reactive oxygen species (ROS) in ME/CFS patients that may stem from inflammatory processes or mitochondrial dysfunction [43]. During prolonged exercise for example even in the healthy situation oxidative stress may reduce Na^+^/K^+^-ATPase activity via glutathionylation [39].

A strong rise in intracellular sodium can activate the sodium-calcium-exchanger (**NCX**) to operate in its reverse mode. Instead of removing calcium the NCX imports calcium to cause calcium overload. The final cellular damage is then due to calcium overload, the paradigm of ischemia–reperfusion damage. This mechanism is well-known to occur in the heart [34–36] and skeletal muscle [44, 45] where abrupt reperfusion follows a brief episode of total ischemia. Ischemia alone in this situation of ischemia/reperfusion situation is too short to cause damage but reperfusion strongly activates the NHE1 and the reverse mode of the NCX to cause damage via calcium overload. The ME/CFS situation is certainly not an ischemia–reperfusion situation where ischemia is total but short and reperfusion is abrupt. What is needed to cause calcium overload is a certain extent of sodium overload only to reverse the transport direction of the NCX. The reversal potential of the NCX does not only depend on sodium concentrations and gradient, but also on calcium concentrations and the membrane potential so that it is not possible to give the sodium concentration at which this occurs.

Sodium overload seems to occur in exercise in ME/CFS suggested by the following findings. Skeletal muscle acidosis is found in ME/CFS patients during or after exercise [23, 26–28, 46]. Proton efflux from skeletal muscle immediately post-exercise was found strongly diminished [46] for a short time only, indicating a transient disturbance of sodium-proton-exchange mechanisms. NHE1 is the most important proton extruder in skeletal muscle. There is no other simple explanation for a transient inhibition of the NHE1 than a high intracellular sodium concentration—accumulated during exercise—as the NHE1 is not directly dependent on ATP but only indirectly via the Na^+^/K^+^ATPase. The latter maintains the sodium gradient at which the NHE1 can work. Sodium and calcium overload could occur in ME/CFS in muscles if exercise is continued under poor energetic conditions.

Does muscular acidosis itself play an important role as a damaging mechanism? Although proton efflux is found decreased immediately post-exercise in ME/CFS there is no severe acidosis and the delay of proton efflux is in the range of less than a minute [46]. It is unlikely that such a short and moderate acidosis could cause a severe disturbance to explain PEM that can last for more than 24 h but calcium overload can do. A big surprise in the early pharmacology of NHE1-inhibition was the recognition that it was not acidosis that damaged muscles but the attempt to get rid of the intracellular protons that finally leads to calcium overload via sodium uptake and overload. In a number of pathological cardiovascular conditions the NHE1 was found upregulated and inhibition of NHE1 conferred strong tissue protection by reducing sodium load and subsequent calcium overload [34–36, 44, 45]. Myocardial fibrosis has been found in some patients with ME/CFS [47]. It causes diastolic (filling) dysfunction to enhance the effect of hypovolemia on preload failure. An upregulated NHE1 is linked with cardiac fibrosis in cardiac diseases, indeed. In experimental cardiovascular medicine cardiac fibrosis is prevented by pharmacological inhibition of (an upregulated) NHE1 [34, 48]. A candidate for stimulating systemic upregulation of NHE1 in ME/CFS is the myocardial infarction associated transcript (MIAT). The latter is found upregulated in ME/CFS patients [49]. MIAT regulates the zinc finger E-box binding homeobox 1 (ZEB1) expression which can cause upregulation of NHE1 [50]. ZEB1 was found upregulated in ME/CFS patients as well [49]. Furthermore, NHE1 activity could be increased via stimulation by alpha1-adrenergic receptor agonists. Since sympathetic tone is increased in ME/CFS (an upregulated) NHE1 may be stimulated by this mechanism [51].

In cardiovascular pathophysiology, NHE1 in the heart is easily upregulated in many pathological situations that are related to a worsened metabolic situation. Since the NHE1 is the most important proton-extruder in skeletal muscle, too, it would really be surprising if it were not upregulated in skeletal muscle in ME/CFS in face of the deep metabolic disturbance. Overall, an enhanced NHE1-activity in skeletal muscle would increase the potential for sodium- and subsequently calcium overload.

The response of PBMCs from ME/CFS patients to a hyperosmotic stressor (high extracellular sodium) in a test measuring impedance was dramatically different from the response observed among the controls so that this test may have the potential as a diagnostic marker of ME/CFS [52]. The hyperosmotic stress applied, which induces cellular shrinking, is a massive challenge for cellular osmo- and volume regulators. NHE1 is most likely the most important volume regulator in leukocytes [53]. As shown before, suspension of human PBMC in exactly the same hypertonic medium indeed induced rapid cellular shrinkage and subsequent activation of NHE1 [53] leading to sodium influx which is followed by influx of water causing repletion of the cellular volume. Intracellular sodium and cell shape (through cellular volume) are certainly determinants of electrical conductance in such an experimental setting measuring impedance. We wonder whether the altered change in conductance in ME/CFS is related to an altered NHE1 activity and potentially secondary consequences arising from sodium overload and potentially calcium overload.

Taken together, we strongly assume that NHE1 activity is enhanced in ME/CFS in skeletal muscles by several mechanisms including increased proton load by an altered muscular metabolism. Both effects would increase cellular sodium load while Na^+^/K^+^-ATPase activity would be decreased because of low ATP levels, insufficient agonistic stimulation and inhibition by ROS, leading to calcium overload as the final damaging mechanism. We assume that the threshold for this ionic disturbance is very much lowered by the disease mechanisms outlined above (Fig. 3) so that it could repeat again and again even under efforts of everyday life to perpetuate the disease as will be explained below. The proximity of the intracellular sodium concentration to this reverse mode threshold of the NCX is the presumed main cause for exercise intolerance in ME/CFS and the potential explanation for the characteristics of a threshold of effort for the induction of PEM (Fig. 3).

### Disturbances of intracellular potassium

Diminished activity of the Na^+^/K^+^-ATPase could affect potassium homeostasis as the Na^+^/K^+^-ATPase takes up two potassium ions in exchange for three sodium ions (Fig. 2). There is evidence for a reduced potassium efflux in exercising muscle in ME/CFS patients which can only be explained by the development of intracellular hypokalemia as a consequence of a diminished activity of the Na^+^/K^+^-ATPase [43]. These authors incriminate ROS to inhibit the Na^+^/K^+^-ATPase [39]. Potassium leaving the cells during excitation via repolarizing potassium channels would not be taken up fast again into the myocytes by the Na^+^/K^+^-ATPase and potassium would be renally excreted resulting in a loss of potassium, mainly intracellular potassium but blood potassium could also be lowered at least at rest. Renal potassium loss may be favored by renal hyperfunction which we have explained above. In line with a diminished activity of the Na^+^/K^+^-ATPase total body potassium was found decreased by about 10% in a group of patients with severe fatigue [54]. In this study, there was a strong inverse correlation of the total body potassium and the total time spent resting as a measure of fatigue and exhaustion. We also observed lower serum potassium to correlate with more severe fatigue and orthostatic dysfunction in ME/CFS patients (unpublished).

The ß_2_AdR agonist salbutamol stimulates the Na^+^/K^+^-ATPase and thereby increases cellular potassium uptake in man to lower plasma potassium as does insulin demonstrating the relevance of the ß_2_AdR for the activation of the Na^+^/K^+^-ATPase and for intracellular potassium [55, 56]. Accordingly, diminished Na^+^/K^+^-ATPase activity by dysfunctional ß2AdR and the other causes mentioned in Table 1 would cause intracellular hypokalemia. Diminished Na^+^/K^+^-ATPase activity may not be the only cause for intracellular hypokalemia. ATP-sensitive potassium channels (KATP) are present on the surface and internal membranes of cardiac, skeletal, and vascular smooth muscle cells (VSMC) and provide a feedback between muscle cell metabolism and electrical activity (Fig. 2). In doing so, they can play an important role in the control of contractility, particularly when cellular energetics are compromised protecting the tissue against calcium overload [57, 58]. Typically, the KATP-channel is closed and not functional at normal ATP levels but opened with ATP depletion. Opening of KATP-channels in a situation of ATP-depletion would cause hyperpolarization to reduce calcium influx to limit contractility and muscle work. While this may reduce the risk of damage it could limit the ability to exercise. Longer lasting opening of KATP channels could lead to significant potassium efflux that could contribute to intracellular hypokalemia as re-uptake of potassium is already reduced by an insufficiently stimulated Na^+^/K^+^-ATPase. Low intracellular potassium has depolarizing effects causing hyperexcitability to increase calcium influx. This may cause cramps and pain at rest to contribute to PEM and myalgia and increase energy consumption at rest which would impair recovery potentially together with a lower blood flow at rest due to the sympathetic vasoconstrictor predominance. Intracellular hypokalemia would finally reduce the driving force for the KATP as a protective mechanism during exercise. A diminished KATP would take away a physiological brake against overexertion to enable a higher muscle performance even when the metabolic situation has already worsened which could bring the patient closer to the threshold of muscular damage. Muscular performance would be incommensurate to the true energetic situation. In other words, ME/CFS patients may perform better than reflected by their true, probably poor energetic situation in skeletal muscle so that they may already perform muscle work at a limit or an energy level which is not tolerable for them (relative overexertion). “Overexertion” can already be said to occur in ME/CFS patients at levels of everyday life exercise which cause the typical PEM.

Promising preliminary effects were reported with the acetylcholine-esterase inhibitor Mestinon™ (Pyridostigmine) presumed to cause constriction of capacity vessels to reduce venous pooling so as to improve cardiac preload [59]. Since the effect of acetylcholine in skeletal muscle includes stimulation of Na^+^/K^+^-ATPase – the effect is presumed to occur and to be limited to resting conditions [39]—one has to consider an alternative possibility namely that the improvement by Mestinon™ occurs via stimulation of the Na^+^/K^+^-ATPase. The pathological ionic changes during exercise—intracellular rise in sodium and loss of potassium—could be corrected much earlier at rest providing faster and better recovery. By stimulating the Na^+^/K^+−^ATPase Mestinon™ could also reduce hypokalemia-induced depolarizations that otherwise lead to cramps and that increase energy consumption at rest. Faster and better repletion of intracellular potassium could restore the driving force of this protective KATP potassium current in critical energetic situations.

### Disturbance of muscular metabolism by calcium overload

Mitochondrial dysfunction is one of the favourite explanations in the literature for fatigue and the pathophysiology of ME/CFS but there is no clear idea about the mechanism [60, 61]. The typical ischemia–reperfusion situation causes mitochondrial dysfunction by cellular calcium overload as a consequence of sodium overload [62, 63]. Mitochondrial dysfunction could play an important role in conjunction with impaired perfusion to explain fatigue and PEM. Calcium overload in the skeletal myocytes could cause cramps or necrosis (probably single cell necrosis if any). The typical ischemia–reperfusion injury usually causes cellular damage and necrosis apart from inducing vascular and mitochondrial dysfunction and insulin resistance. We assume that the damage caused in ME/CFS mainly remains in the functional range as a stronger disturbance would make the patient stop working immediately while ischemia–reperfusion experiments are performed in anesthetized animals. However, there is even recent evidence for minimal structural damage of skeletal muscles in ME/CFS by the finding of actin network proteins, talin-1 and filamin-A in circulating extracellular vesicles [64]. The latter are released from damaged or stressed cells with cellular content, such as proteins, and circulate in the bloodstream.

Does skeletal muscle insulin resistance occur in ME/CFS in consequence of ischemia–reperfusion like damage to skeletal muscles? High insulin levels have been reported in ME/CFS patients [65]. We assume that the elevated insulin levels are caused by insulin resistance (as in the metabolic syndrome) mainly in the muscles which would further disturb muscular metabolism. Importantly, the Na^+^/K^+^-ATPase is also stimulated by insulin (Table 1). Ischemia–reperfusion injury has been shown to cause insulin resistance by muscular calcium overload [66, 67]. Insulin-resistance, in turn, has been shown to worsen the ischemia–reperfusion damage [68–70]. Hence, there is a potential other vicious cycle that could maintain the state of ME/CFS. Previous exercise negatively affected exercise 24 h later with regard to physical performance and arterial lactate (rise) in ME/CFS patients while the opposite occured in healthy subjects [38] in line with a deterioration of the muscular metabolic situation by exercise. The expectation is that insulin sensitivity is decreased in the affected muscles in ME/CFS but (primarily) normal in adipose tissue (at least for a longer period) where higher insulin levels could favor fat deposition. Insulin resistance (together with mitochondrial dysfunction) may only represent a broader disturbance of cellular metabolism induced by calcium overload. Cultured human skeletal muscle cells from patients with ME/CFS showed impaired glucose uptake upon stimulation proximal to AMPK-activation [25] for which insulin resistance could be a theoretical explanation. Apart from other metabolic effects insulin resistance could further raise intracellular sodium levels in skeletal muscles because the importance of the insulin-independent but sodium-dependent glucose transporters (SGLT) could increase to compensate for the diminished activity of the insulin-dependent but sodium-independent glucose transporters (GLUT).

Interestingly, MELAS, a severe genetic mitochondrial disease, which affects about ~ 1 in 5000 (~ 65k US patients) mainly affects brain, heart and skeletal muscles and like all mitochondrial diseases is associated with fatigue, elevated lactate levels and with defects in nitric oxide (NO) signaling, ED and impaired blood flow [71]. In ME/CFS mitochondrial dysfunction is acquired, milder, functional, and most likely restricted to skeletal muscle while in MELAS mitochondrial dysfunction in the CNS leads to CNS symptoms including stroke-like episodes, headaches, and seizures. Thus, MELAS and ME/CFS share mitochondrial dysfunction with elevated lactate levels, fatigue, ED and perhaps also insulin resistance as has been outlined in the paragraph above. MELAS clearly demonstrates that mitochondrial dysfunction can induce ED.

### Vascular (endothelial) dysfunction induced by calcium overload

The typical ischemia/reperfusion situation causes microvascular dysfunction [72–76]. Effects on endothelial cells known from hypoxia/reoxygenation experiments are a strong increase in endothelial contraction increasing endothelial permeability [74] potentially leading to muscular swelling. If this mechanism of sodium overload leading to detrimental calcium overload was present in the endothelium of the muscle it could cause endothelial dysfunction repeatedly with every “excessive” physical effort to fix the state of endothelial dysfunction in the skeletal muscle favoring sympathetic vasoconstriction.

Molecular mechanisms underlying ischemia–reperfusion injury in striated muscles involve the production of reactive oxygen species (ROS) [77]. In ME/CFS, ROS released as a result of the presumed calcium overload may also be involved as a mechanism damaging myocytes and endothelium contributing to vascular and endothelial dysfunction and to an inhibition of the Na^+/^K^+^ATPase.

### Possible scenario of altered ion homeostasis at rest on mitochondrial function

After having considered the consequences of high sodium loading above the threshold where the NCX (of the sarcolemma) changes from forward to reverse mode and importing calcium to cause calcium overload we now explain the ionic changes that could take place at sodium concentrations below this threshold where the NCX would still operate in the forward mode. This is probably the case most time at rest and perhaps still during very moderate levels of exercise in ME/CFS. Even in the forward mode a rise in intracellular sodium would already raise intracellular calcium by a decrease in the sodium gradient lowering the driving force of the exchanger leaving more calcium in the cells, but this would occur in a controlled and rather linear manner. By the way, this is the mode of action how digoxin raises calcium in the heart to cause its positive inotropic effect (rise in intracellular sodium is followed by a rise in calcium). By contrast, reversal of the transport direction of the NCX (reverse mode) by high intracellular sodium leads to a sudden and steep rise in intracellular calcium that can easily get out of control and lead to calcium overload. This occurs at levels of exercise that cause PEM according to our hypothesis.

Cytoplasmatic calcium stimulates cytosolic metabolism. What about mitochondrial calcium which drives mitochondrial metabolism? The mitochondrial situation is much more complicated. There are two opposing forces on mitochondrial calcium in this situation of elevated sodium. A higher cytoplasmatic calcium would also tend to raise mitochondrial calcium. However, since cytoplasmatic sodium rises and since cytoplasmatic sodium is the driving force for the mitochondrial NCX – named NCLX—which is different from the NCX of the sarcolemma [78], a rise in intracellular sodium tends to lower mitochondrial calcium (Fig. 2). It is difficult to predict what the result of these two opposing forces on mitochondrial calcium would be. Would it be higher than normal or lower than normal? What can be said is that the relative rise in cytoplasmatic calcium would always be higher than that of the mitochondrial calcium and therefore be disproportionate to the latter. A dysbalance between cytoplasmatic and mitochondrial calcium may occur with potential consequences for mitochondrial versus cytosolic metabolism: is stimulation of glycolysis in cytoplasm therefore stronger than that of oxidative phosphorylation in the mitochondria? Does this lead to a situation of anaerobic glycolysis even at rest? To be more specific, ME/CFS patients do not only show elevated lactate levels after exercise; a recent paper shows that a group of ME/CFS patients (about half) has already elevated lactate levels at rest and these correlate with PEM [79]. During exercise blood flow to skeletal muscle can increase 20 to 30-fold reflecting the high metabolism during exercise. While a mismatch between ATP demand and generation is highly likely to occur during exercise in ME/CFS the reported anerobic glycolysis at rest is unlikely to be due to such a mismatch as the metabolic need is so much lower at rest. This finding of elevated lactate levels at rest could be due to a dysregulation of metabolism by disparate calcium signals in cytoplasm versus the mitochondrium so that their respective metabolisms could get uncoordinated (uncoupling between cytosolic and mitochondrial metabolism). It cannot be predicted on theoretical grounds whether mitochondrial calcium is lower or higher than normal at rest. This might even be situation dependent or different between individuals. In the following we want to speculate on what could happen if mitochondrial calcium were lower than normal most of the time at rest. A lower mitochondrial calcium at rest may lower oxidative phosphorylation creating unfavorable conditions for starting exercise (fatigue at rest to be distinguished from fatigability during exercise). The level of mitochondrial calcium may suddenly change with exercise when a further rise in intracellular sodium will abruptly change the mode of the NCX of the sarcolemma into the reverse mode to suddenly cause cellular and mitochondrial calcium overload. This may hit a mitochondrium potentially sensitized to calcium. Thus, even relatively moderate but sudden increases in calcium could have detrimental effects. Is mitochondrial calcium low at rest (potentially causing hypersensitivity to calcium) but gets abruptly high during exercise (overload) to cause functional mitochondrial damage? Does mitochondrial calcium overload then cause temporary desensitization to calcium while mitochondrial calcium concentration may be low again at rest for reasons explained above. Both effects together could cause a substantial deficit in calcium stimulation of the mitochondrium at least for a while with serious consequences for mitochondrial metabolism (energy crisis). With a time delay low mitochondrial calcium at rest could then again cause hypersensitivity to calcium improving energy production but creating the conditions for the next hit by effort-induced sodium overload and subsequent calcium overload thus contributing to the perpetuation of the disease.

We assume calcium overload is the main mechanism of PEM, exercise intolerance and of more severe relapses. In ME/CFS patients, intracellular sodium is presumably either close to the threshold for reverse mode NCX activation already at rest or, alternatively, rises rapidly during exercise to reach the level at which the NCX changes into the reverse mode to cause calcium overload (Fig. 3). Thus, even at moderate levels of exercise of everyday life this threshold may be easily crossed causing calcium overload and PEM. The sodium concentration threshold for reverse mode NCX activation may explain the existence of an effort threshold for the induction of PEM.

### Energy waste in skeletal muscle by overstimulation of thermogenesis?

It is widely assumed that a cellular energetic disturbance in skeletal muscle is present in ME/CFS that is mainly resulting from a mitochondrial dysfunction as we outline above. The prevalent cardiovascular situation with low preload and high sympathetic (vasoconstrictor) tone should reduce skeletal muscle perfusion. Both disturbances (i.e. mitochondrial dysfunction & reduced skeletal muscle perfusion) together could then finally lead to a precarious energetic situation.

**Fig. 4:**
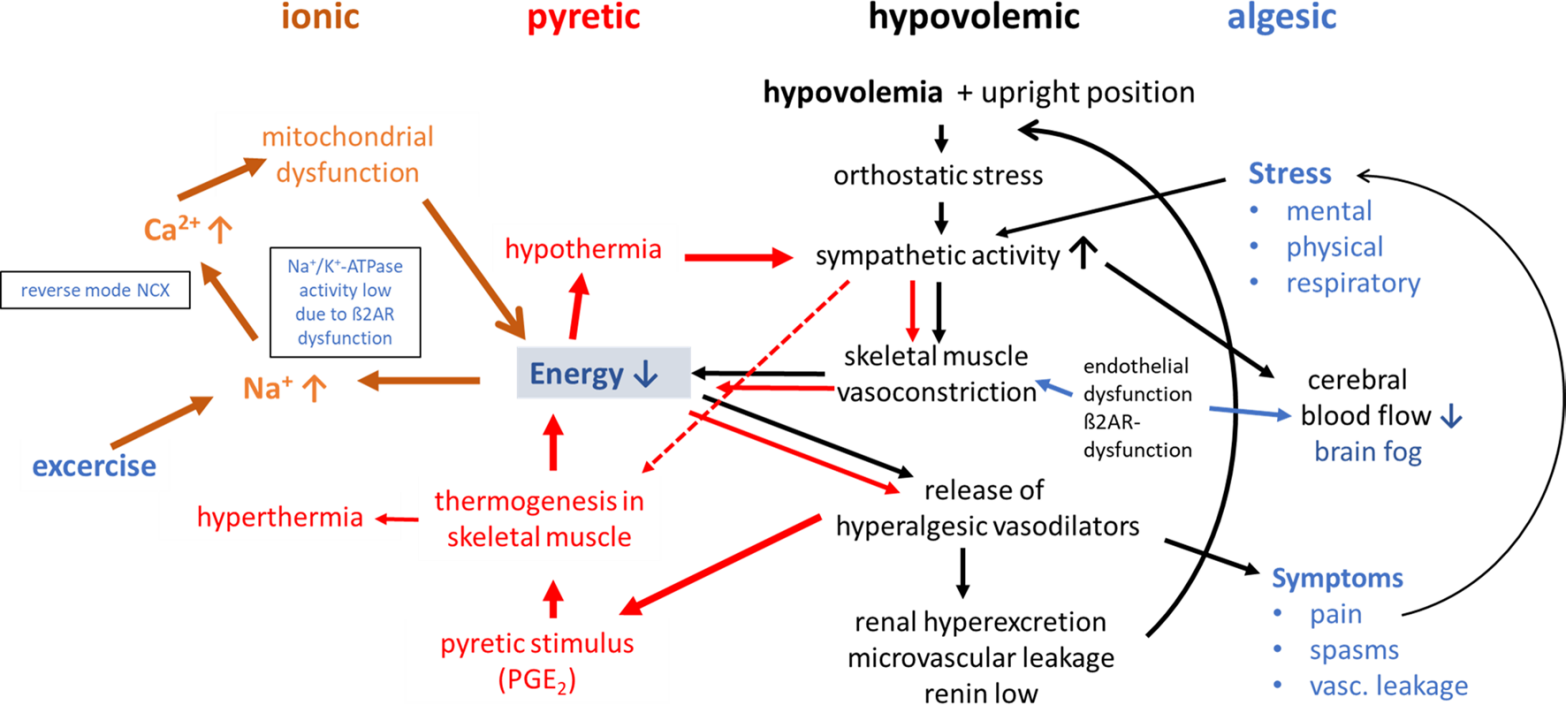
Exercise, Hypovolemia, Stress and Disturbed Thermoregulation in ME/CFS: A Self-stabilizing Interplay between Vicious Circles

Skeletal muscle is the most important organ for heat production in man at basal metabolic rate as well as at stimulated metabolic conditions, like non-shivering (NST; uncoupling SERCA) and shivering thermogenesis [80]. Since many ME/CFS patients complain about fever, sensations of heat or cold and intolerance to heat and/or cold the question arises whether disturbed thermoregulation is of potential relevance for the skeletal muscle pathophysiology and the energetic situation in skeletal muscles. Hyperthermia could be explained by catecholamines and, particularly, the pyretic effect of PGE_2_ (central stimulatory effect) which we assume either is released directly from skeletal muscle or indirectly via bradykinin-stimulated cyclooxygenase not only during exercise but also in orthostatic stress and mental efforts as we postulate in our first hypothesis paper [6]. In ME/CFS, fever stimulating skeletal muscle metabolism could waste energy by heat production thereby even more worsening the already precarious energetic situation. This, in turn, should again favor compensatory release and spillover of inflammatory vasodilators including PGE_2_ to further stimulate thermogenesis resulting in a potential vicious (“pyretic”) cycle (Fig. 4). Energy depletion could finally cause hypothermia. Would sensation of cold, similar to environmental cold—cold stress—then also raise sympathetic tone to cause (more) vasoconstriction and potentially (further) stimulate thermogenesis as illustrated in Fig. 4? Such a vicious cycle could be of relevance in particular for severe ME/CFS where it may considerably delay or hinder recovery of the energetic situation by continuously wasting energy by thermogenesis. Inappropriate stimulation of thermogenesis, in addition to exercise and stress, should therefore be added to the list of potentially detrimental mechanisms in skeletal muscle pathophysiology of ME/CFS.

Above we describe how following exercise sodium overload could lead to calcium overload thereby causing mitochondrial, energetic and endothelial dysfunction in skeletal muscle. We assume that the threshold for this pathomechanism becomes very low and falls into the range of everyday life physical activities as soon as the disease is established so that it is repeatedly triggered to perpetuate the disease. Is it possible that at this lowered threshold such ionic derangements, which we assume will finally cause PEM and exercise intolerance, are already induced by the combination of orthostatic or mental stress with skeletal muscle thermogenesis independent of actual physical exercise by the mechanisms described here, once the disease has become manifest (Fig. 4)? Orthostatic stress seems to be more important than assumed so far and manifests as a decrease in cerebral blood flow not only when standing up but in severe cases already in a sitting position [8, 9] so that it seems to be a basic stressor to which in real life all other forms of stress are additive. Is the combination of stress of any cause together with stimulation of skeletal muscle thermogenesis causing energy consumption a pathophysiological equivalent in skeletal muscle to low or moderate levels of physical exercise that can induce PEM via the mechanisms described in this paper?

## Discussion

**Fig. 5:**
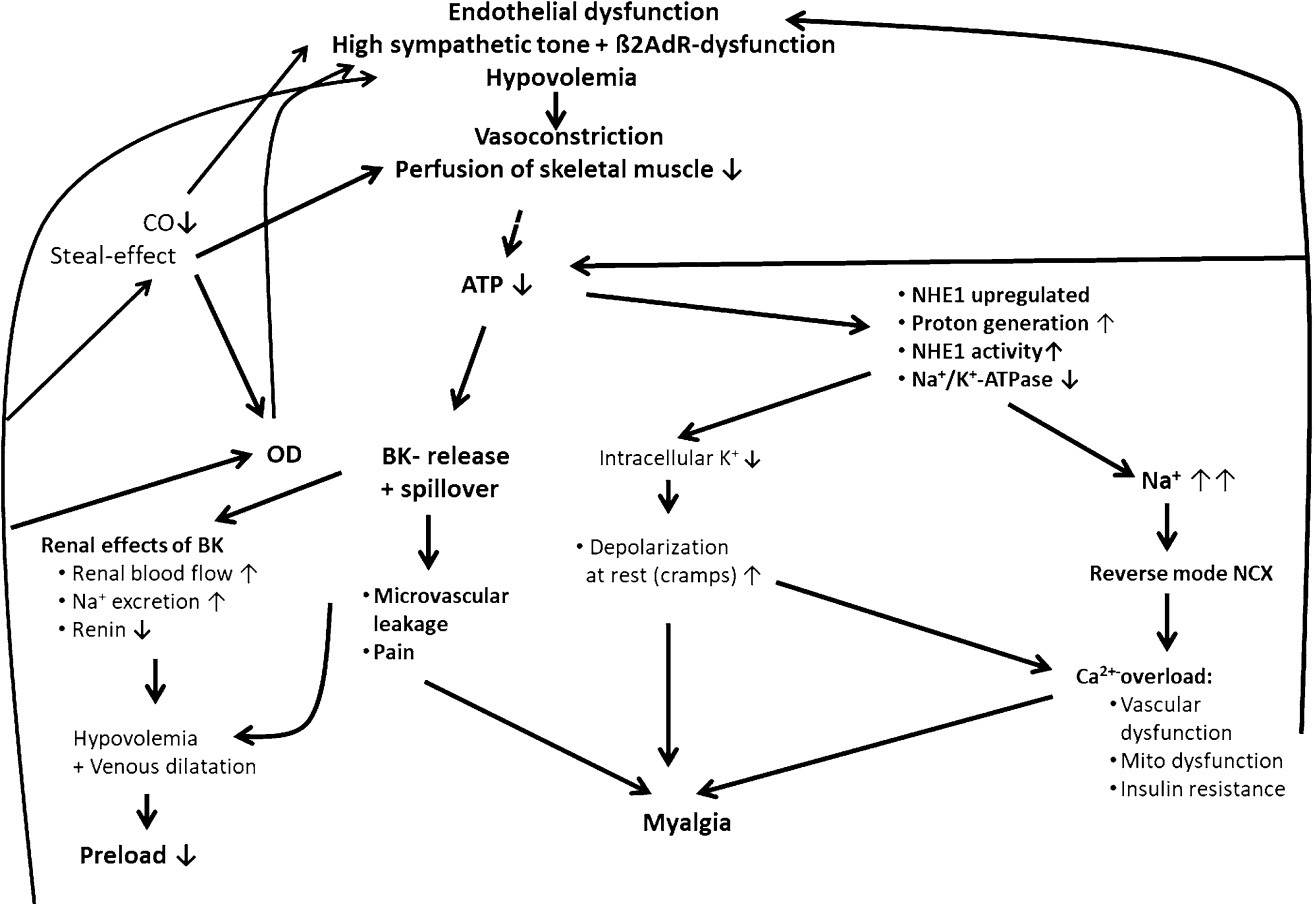
Hypothetical pathomechanisms in skeletal muscle and in cardiovascular system in ME/CFS explaining fatigue and myalgia. Bradykinin (BK), Cardiac output (CO), Orthostatic dysfunction (OD)

In our first hypothesis paper we identified high sympathetic tone (stress), hypovolemia (preload failure), endothelial or vascular dysfunction for which ß2AdR dysfunction could be the most important cause, and a still enigmatic energetic disturbance in skeletal muscle as critical factors in the pathophysiology [6]. Hypoperfusion in skeletal muscle leads to the release and spillover of metabolically activated (hyper) algesic vasodilators as a compensatory response strongly favored by a poor metabolic situation (Fig. 5). The appearance of these excessively produced vasodilators with algetic properties in the system circulation can explain hypovolemia, the renin paradoxon and many symptoms of ME/CFS. In the present hypothesis paper, we tried to explain the causes of the energetic disturbance in skeletal muscle which we assume strongly favors the release of algesic vasodilators and which is the major cause of exercise intolerance. A higher metabolic production of protons due to the metabolic disturbance leads to cellular sodium loading by the NHE1 in the process of proton extrusion while the capacity of the Na^+^/K^+^-ATPase to remove sodium is reduced for several reasons. Among those ß2AdR dysfunction may be the most important one. Insufficient Na^+^/K^+^-ATPase activity leads to a rise in intracellular sodium and to intracellular hypokalemia. At high intracellular sodium concentration, the NCX of the sarcolemma can change its transport direction into the reverse mode importing calcium instead of exporting it which can lead to calcium overload. The resulting damage can affect metabolism particularly the mitochondria in skeletal muscles, cause local endothelial dysfunction and insulin resistance. The functional damage induced by calcium overload causes the poor energetic situation as well endothelial dysfunction in skeletal muscle. The proximity of the intracellular sodium concentration to the threshold for reverse mode activation of the NCX in skeletal muscle or alternatively a very rapid rise in sodium during even moderate levels of exercise causes this threshold to be crossed rapidly during exercise in ME/CFS (Fig. 3). As just explained above the combination of stress with stimulation of skeletal muscle thermogenesis could be a pathophysiological equivalent in muscle to low or moderate levels of physical exercise.

Spontaneous recovery remains rare in ME/CFS patients regardless of disease severity [81] because the patient is caught in a self-stabilizing network of these vicious circles or dysregulations (Figs. 4 and 5). A mechanical analogy to this interplay shown in Fig. 4 is a gear box in which moving a single gearwheel sets the whole gearing mechanism in motion. Based on these hypotheses we try to explain the pathomechanisms and the sequence of events differentiating between precipitating and perpetuating mechanisms and risk factors in the following section.

### Precipitating mechanisms

#### Infections

The etiology of postinfectious fatigue syndromes is yet unresolved. Apart from potentially inducing autoimmunity, infections may initiate hypovolemia by fever or diarrhea or induce a state of vascular leakage raising a potentially high sympathetic tone (stress), cause endothelial damage and dysfunction and cardiovascular and respiratory stress. The latter also raises sympathetic tone via stimulation of peripheral chemoreceptors. Viral infections could affect the muscles, particularly its endothelium or vessels causing endothelial dysfunction creating the conditions for sympathetic vasoconstriction. Covid-19 is a current example for a severe viral infection triggering ME/CFS. At least two requirements postulated here seem to be met in Covid-19 infections: the endothelium seems strongly affected causing a number of cardiovascular complications, and hypoxia causes respiratory hypoxic stress [82]. A third potential mechanism will be outlined below.

### Non-infectious mechanisms

A fraction of the ME/CFS patients having no history of severe viral infection experiences the beginning of ME/CFS in close temporal relationship with overexertion [83]. This is most likely unrelated to mechanisms of infection or autoimmunity and therefore of high pathophysiological interest. Overexertion can lead to hypovolemia and a high sympathetic tone. Exhaustive exercise could foster mitochondrial disturbances in skeletal muscles [84]. Overexertion could induce the same disturbances in sensitive otherwise healthy subjects as outlined above in ME/CFS, particularly skeletal muscle calcium-overload with mitochondrial, metabolic and vascular (endothelial) dysfunction although the threshold for the (first) induction of this kind of damage should be very high in the healthy state. Therefore, ME/CFS patients with overexertion as a likely cause may have unknown predispositions for this disturbance. As soon as this damage would have occurred in a severe manner the threshold for it to occur again could be very much lowered in the presence of a high sympathetic tone, hypovolemia and dysfunctional ß2AdR so that it could repeat at much lower levels of exercise, even during activities of everyday life, re-inducing mitochondrial, metabolic and vascular dysfunction again and again to perpetuate the disease. Upregulation of the NHE1 would increase the potential for repeated sodium- and calcium overload.

In our first hypothesis paper [6] we elaborated on the frequent association of OI, POTS and ME/CFS and hypothesized that causality could be bidirectional. ME/CFS could aggravate orthostatic dysfunction by causing hypovolemia and preload failure. On the other hand, we believe that primary severe orthostatic dysfunction could initiate ME/CFS by orthostatic stress causing excessive vasoconstriction and desensitization of ß-adrenergic receptors, particularly of the ß2AdR leading to the hypothetical pathophysiological changes in skeletal muscle as explained above. Meanwhile the appraisal of the role of orthostatic dysfunction may have been strengthened by several recent publications showing that orthostatic dysfunction in ME/CFS is even present in a sitting position so that it can be considered as the basic and permanent stressor in almost all human activities in the awake state [8, 9]. Orthostatic vascular regulation and vascular adaptation to exercise require a high level of coordinated physiological regulation. Many disturbances seem possible leading to orthostatic dysfunction with the consequence of raising sympathetic (vasoconstrictor) tone and desensitizing ß-adrenergic receptors which is supposed to worsen skeletal muscle blood flow and which has been shown to decrease cerebral blood flow [8, 9].

The possibility of autoantibodies against other structures involved in vascular regulation or volume regulation has to be considered, particularly in case of a postinfectious onset of the disease.

### Perpetuating mechanisms

The infection-induced primary causes of hypovolemia and endothelial dysfunction may well disappear but once a state of muscular energetic and vascular dysfunction was initiated the threshold for a repeated calcium overload would be very low and could occur with activities of everyday life to maintain a state of metabolic disturbance and of endothelial or vascular dysfunction.

Excessive release and spillover of hyperalgesic vasodilators, like bradykinin, which are generated in the process of functional sympatholysis in the affected muscles as a consequence of dysfunctional ß2AdR receptors in the presence of a high sympathetic tone and favored by a poor energetic situation could maintain a state of hypovolemia as outlined in our first hypothesis paper [6]. Orthostatic stress seems to be more important than assumed so far; in severe disease it is present already in a sitting position [8, 9]. Thermogenesis in skeletal muscle may be stimulated by PGE_2_ and catecholamines to cause a waste of energy in skeletal muscle further worsening the precarious energetic situation. Energy depletion may lead to hypothermia causing cold stress to further stimulate thermogenesis giving rise to a vicious cycle in which energy loss is even enhanced.

Muscular insulin resistance and degeneration of unmyelinated fibres [40] may contribute to diminished Na^+^/K^+^-ATPase activity because insulin and cGRP, which is released from these nerves, stimulate the Na^+^/K^+^-ATPase [32] (Table 1). A chronically high sympathetic tone may lead to vascular hypertrophy enhancing sympathetic vasoconstriction in skeletal muscles favoring metabolically stimulated release of algesic vasodilators.

Several noncoding RNAs targeting vascular and muscular function were found dysregulated in ME/CFS suggesting an important role in their regulation [16, 49, 85]. A recent study showed different miRNA signatures in response to PEM induction allowing classification of ME/CFS patients into four clusters associated with symptom severity [85].

### Potential risk or predisposing factors

A dysbalance between the RAAS, which causes volume repletion, and its physiological antagonistic system, the kallikrein-kinin-system (KKS), which favors volume loss by sodium and water excretion, could favor ME/CFS. In cardiovascular diseases, particularly hypertension, there is a tendency for a predominance of the RAAS over the KKS and for hypervolemia as a factor causing hypertension. It remains to be investigated whether the opposite is true for ME/CFS, namely a predominance of the KKS over the RAAS favoring hypovolemia. In Covid-19 this could be the case. There is evidence that the RAAS activity is impaired by the high expression of ACE_2_ while the activity of the KKS is increased by an enhancement of the sensitivity of the bradykinin receptor to bradykinin [86]. Altogether, at least three mechanisms postulated here and in our previous paper seem to be met to explain the risk for developing ME/CFS after Covid-19, including endothelial affection, stress (respiratory and cardiovascular) and a potential dysbalance between RAAS and KKS.

Degeneration of unmyelinated fibres may not only be a perpetuating factor occurring as a consequence of ME/CFS but may be present before as a risk factor and contribute to diminished Na^+^/K^+^-ATPase- and vasodilator activity by a deficit in cGRP [40].

Ehlers-Danlos syndrome, which can also affect the large blood vessels, is a known risk factor for ME/CFS [87] which is not understood. One of several factors may be the inability of contracting distended large capacitance vessels, which may limit the possibility of raising the circulating blood volume in hypovolemia. Pain and chronic stress may be involved. Cranio-cervical instability could cause carotid body chemoreceptor irritation leading to a strong sympathetic stimulation as hypothesized previously [6].

Considering all the potential predisposing factors we think that in the presence of a high sympathetic tone dysfunction of the ß2AdR could play an essential role (conditio sine qua non) in the etiology of ME/CFS although secondary and rather complex mechanisms have to get involved and to interact, particularly in the chronification of the disease (Figs. 4 and 5). Dysfunction of the ß2AdR by autoantibodies, mutations and desensitization by chronic stress would favor vascular dysfunction and cause insufficient stimulation of the Na^+^/K^+^-ATPase at least as a risk factor together with other precipitating factors. There may be numbers of different, still unknown predispositions.

### Outlook

According to our hypothesis dysfunctions of the ß2AdR or post-receptor mechanisms, of the NHE1, the Na^+^/K^+^ATPase, the NCX, the RAAS and the KKS could be causally involved in ME/CFS and should therefore be further investigated. This concept offers potential novel strategies for the treatment of this debilitating disease.

## Data Availability

Not applicable.
